# Supplementary material for: Contemporary patterns in kidney graft survival from donors after circulatory death in the United States
Source: PLoS One. 2020 May 29;15(5):e0233610. doi: 10.1371/journal.pone.0233610 (PMC7259576; doi:10.1371/journal.pone.0233610)
Supplement: Attachment — Submitted filename: Response to Reviewers_5.2.20.pdf [file pone.0233610.s002.pdf]

University of Washington
Division of Transplant Surgery, Department of Surgery, University of Washington
1959 NE Pacific St., Box 356410, Seattle, WA 98195-6410
lensasi@uw.edu

May 2, 2020

Dear Dr. Dor and the PLOS ONE editorial team,

Thank you again for your interest in this manuscript and the opportunity to improve the piece in response to reviewer comments. As requested, we have revised the manuscript to reflect these comments as detailed below. We have also made some small changes to improve language and flow.

Thank you again for your consideration.

Sincerely,
Lena Sibulesky, MD

--
Response to editorial and reviewer comments:

Academic editor:
Thank you for making the changes to your paper as requested. The MS has significantly improved thanks to this. I would like to propose to add reviewer 1's suggestion to the discussion. Provisionally accepted pending the minor revision

Thank you, we have modified to include Reviewer 1's comments as below.

Reviewer #1: Thank you for making the changes suggested by the second reviewer. The large sample of DBD and DCD donors as well as the interesting outcomes, contribute hugely to the value of this paper.

After reading your publication, members of the transplant community will be questioning whether they should be accepting more DCD kidneys. Can I ask you to add your thoughts on this issue and how you may have changed your practice following these outcomes please?

As clinicians, we do believe that this study and others encourage us to consider accepting DCD kidney grafts more readily if KDPI is high but other graft characteristics are favorable. We have added this consideration to the discussion.

Reviewer #2: I want to thank the authors for the latest changes to the paper. The current version has a clear and important message. I have no further comments.

We greatly appreciate the reviewer's comments, which we believe have enhanced the manuscript.
