# Supplementary material for: Contemporary patterns in kidney graft survival from donors after circulatory death in the United States
Source: PLoS One. 2020 May 29;15(5):e0233610. doi: 10.1371/journal.pone.0233610 (PMC7259576; doi:10.1371/journal.pone.0233610)
Supplement: Attachment — Submitted filename: DCD_response to reviewers_1.28.20.docx [file pone.0233610.s001.docx]

University of Washington

Division of Transplant Surgery, Department of Surgery, University of Washington

1959 NE Pacific St., Box 356410, Seattle, WA 98195-6410

[lensasi@uw.edu](mailto:lensasi@uw.edu)

January 28, 2018

Dear Dr. Frank and the PLOS ONE editorial team,

Thank you for the opportunity to re-submit this manuscript. We greatly appreciate the reviewer comments and have improved the manuscript with their feedback in mind. Most notably, we now extend the duration of follow-up by including data that has been released in the time since original submission and report a secondary analysis using the same methodology for an earlier cohort to support more direct comparisons across eras. Similar to our primary analysis, we found no difference in recipient survival between types of deceased donor kidneys for this secondary analysis. In light of this finding, we also now expand on our discussion of how our mixed effects model may also have contributed to differences seen between our study and prior work. We feel that this enriches the report and suggests additional avenues for future investigation. We also now include additional recipient variables in our adjusted analysis to further limit confounding. Finally, we made small changes in the wording to improve overall flow. Major changes are highlighted in red in the revised manuscript.

Thank you again for your consideration.

Sincerely,

Lena Sibulesky, MD

--

**Editorial Staff:**

i) We note that you have indicated that data from this study are available upon request. PLOS only allows data to be available upon request if there are legal or ethical restrictions on sharing data publicly. For information on unacceptable data access restrictions, please see http://journals.plos.org/plosone/s/data-availability#loc-unacceptable-data-access-restrictions.

* In your revised cover letter, please address the following prompts:

If there are ethical or legal restrictions on sharing a de-identified data set, please explain them in detail (e.g., data contain potentially identifying or sensitive patient information) and who has imposed them (e.g., an ethics committee). Please also provide contact information for a data access committee, ethics committee, or other institutional body to which data requests may be sent.

b) If there are no restrictions, please upload the minimal anonymized data set necessary to replicate your study findings as either Supporting Information files or to a stable, public repository and provide us with the relevant URLs, DOIs, or accession numbers. Please see http://www.bmj.com/content/340/bmj.c181.long for guidelines on how to de-identify and prepare clinical data for publication. For a list of acceptable repositories, please see <http://journals.plos.org/plosone/s/data-availability#loc-recommended-repositories>.

Data used in this study is managed by the Scientific Registry of Transplant Recipients. It is publicly available upon request at: <https://www.srtr.org/requesting-srtr-data/data-requests/>

c) We will update your Data Availability statement on your behalf to reflect the information you provide.

ii)  Thank you for stating the following in the Acknowledgments Section of your manuscript:

The interpretation and reporting of these data are the responsibility of the authors and in no way should be seen as an official policy of or interpretation by the OPTN or the U.S. Government. Dr. Butler is supported by a training grant from the NIDDK (5T32DK007467-33).

We note that you have provided funding information that is not currently declared in your Funding Statement. However, funding information should not appear in the Acknowledgments section or other areas of your manuscript. We will only publish funding information present in the Funding Statement section of the online submission form.

* Please remove any funding-related text from the manuscript and let us know how you would like to update your Funding Statement. Currently, your Funding Statement reads as follows:

The author(s) received no specific funding for this work.

We have removed any reference to funding from the manuscript. The updated funding statement should read:

Dr. Butler is supported by a training grant from the NIDDK (5T32DK007467-33).

2. Please remove your figures/ from within your manuscript file, leaving only the individual TIFF/EPS image files.  These will be automatically included in the reviewer’s PDF

This has been corrected.

**Academic Editor**:

This MS is of interest to the tx community, showing equivalent survival of DCD vs DBD kidney transplantation.
Reviewers do highlight some issues that would need to be addressed in detail. Especially, some additional outcomes are missing that would be important to include.

Below, we include a point-by-point response to reviewer comments including regarding alternative outcomes.

**Reviewer #1:**

1. Could I ask you please if you have any data on the type of DCD donor eg controlled or uncontrolled or Maastricht category? If yes, did you do any analysis according to these? If not, please state in the paper that no such data were available.

We have now clarified that all donations in the US are controlled (Maastricht category III). [Introduction, paragraph 2]

2. Do you have any data on delayed graft function to add to the study?

We agree that delayed graft function would be a valuable outcome. However, because definitions of delayed graft function vary between transplant centers and reporting is heterogenous, we did not believe that the current dataset offered sufficiently robust information to include this outcome for the current analysis.

3. Please add the current practice followed in the US with respect to DCD kidney donation:
a. How many minutes is the “waiting period” (time after cessation of cardiorespiratory function before death is declared)?

We now describe the 2-5 minute “waiting period” before declaration of death for DCD donors. [Introduction, paragraph 2]

b. How long do you wait before standing down if there is no cessation of cardiorespiratory function? Does this practice very between US centers?

We now include information about what is typically a 1-hour cut-off time after withdrawal of life-supporting treatment in the US. [Introduction, paragraph 2]

**Reviewer #2:**

This paper describes the association of DCD versus DBD donor status with all cause graft loss after kidney transplantation in a recent US-cohort of transplants performed between April 2014 and September 2017. The authors found no association of DCD status with graft loss.

As many transplant professionals perceive DCD kidneys as inferior and the KDRI is negatively impacted by the DCD donor status this paper has a potentially important message for the acceptance of offered kidneys and allocation policy making. The paper confirms recent papers in outcomes in other countries showing similar outcomes in DCD transplantation as compared to DBD transplantation. Overall the central message is clear and relevant. I do feel that somewhat more effort could have been made to corroborate the findings.

Some comments:

- The median follow up in the study is below one year, thus effects of donor status on longer term outcome may be missed. Follow up is short due to the study design in which the authors aim to analyse a recent cohort. As it has been suggested that DCD donor status negatively impacts early outcome and this is compensated by improved long term outcome, the short term outcome described in this study is probably highly relevant. However, as the authors explain their findings by improved management in DCD transplantation, it may be helpful to actually demonstrate that this short term outcome has improved compared to earlier cohorts.

We have now extended the follow-up period for our cohort as updated data was available in the time since initial submission. We also include a secondary analysis for an earlier cohort in order to support more direct comparisons across eras. We chose to define this earlier cohort with a longer follow-up time to assess whether our choice of model (a mixed cox proportional hazard model) may also contribute to differences that we see between our analyses and prior studies.

- Unfortunately data on other short term outcomes such as delayed graft function or primary non function are not given. Insight into these parameters would be helpful to explain the findings of the study. Again a comparison with earlier cohorts would make the findings more robust.

As noted in response to Reviewer #1’s comments above, we agree that delayed graft function is an outcome of general interest. We decided not to pursue this outcome because we did not find reports in our dataset to be sufficiently consistent to support validity.

- While all cause graft loss as given in the study is the outcome of primary interest, a separate analysis of death censored graft survival may give some additional insight into changing patterns over time.

We decided to not include death-censored graft loss for this analysis because we find it difficult to reliably disentangle what is often a complex relationship between death and deteriorating graft function. For example, failing graft function may nonetheless lead to recipient death before frank graft failure can be reported. We believe that this approach aligns with existing work such as in Rao et al. and Zhong et al.

Minor points:
- A recent study on the Dutch DCD experience confirming the equivalence of graft survival in DCD and DBD kidney transplantation would seem a useful reference. (Schaapherder et al. EClinicalMedicine. 2018 Oct 9;4-5:25-31)

We appreciate the suggestion and now reference this study. [Discussion, paragraph 2]

- The following is stated in the introduction: “A requisite two to five-minute “waiting period” after cessation of cardiorespiratory function and before death is declared can cause systemic hypotension..” “Can cause systemic hypotension” is a somewhat awkward description of the risks after after cessation of circulation.

We have clarified this language. [Introduction, paragraph 2]
